# Genetics‐based dynamic systems model of canopy photosynthesis: the key to improve light and resource use efficiencies for crops

**DOI:** 10.1002/fes3.74

**Published:** 2016-01-04

**Authors:** Qingfeng Song, Chengcai Chu, Martin A. J. Parry, Xin‐Guang Zhu

**Affiliations:** ^1^ CAS Key Laboratory for Computational Biology and State Key Laboratory of Hybrid RicePartner Institute for Computational BiologyChinese Academy of SciencesShanghai200031China; ^2^ The State Key Laboratory of Plant Genomics and National Center of Plant Gene Research (Beijing)Institute of Genetics and Developmental BiologyCASBeijing100101China; ^3^ Lancaster Environment CentreLancaster UniversityLancasterLA1 4YQUK

**Keywords:** Canopy photosynthesis, design crop systems, genetics‐based model of canopy photosynthesis, heterogeneity, microclimates

## Abstract

Improving canopy photosynthetic light use efficiency instead of leaf photosynthesis holds great potential to catalyze the next “green revolution”. However, leaves in a canopy experience different biochemical limitations due to the heterogeneities of microclimates and also physiological parameters. Mechanistic dynamic systems models of canopy photosynthesis are now available which can be used to design the optimal canopy architectural and physiological parameters to maximize CO_2_ uptake. Rapid development of modern crop genetics research now makes it possible to link such canopy models with genetic variations of crops to develop genetics‐based dynamic systems models of canopy photosynthesis. Such models can guide marker‐assisted breeding or genomic selection or engineering of crops to enhance light and nitrogen use efficiencies for different regions under future climate change scenarios.

## Introduction

Photosynthesis is the primary determinant of plant biomass. Canopy photosynthesis is the sum of photosynthetic rates for all photosynthetic tissues (e.g., leaves, stems ears) within the canopy. Since the room to further increase crop harvest index is small, improving canopy photosynthesis and hence biomass production are now widely recognized as a major avenue to increase crop yields. A number of options to increase canopy photosynthesis has been proposed, such as optimizing Rubisco kinetic parameters, increasing the speed of recovery from photo‐protective states, decreasing the antenna size of photosystems, etc. (Zhu et al. 2010; Parry et al. 2013; Carmo‐Silva et al. 2015; Lin et al. 2014). There is existing substantial heritable variation in photosynthetic traits (see for example Driever et al. 2014) and biotechnological targets (Parry et al. 2011; Ort et al. 2015) that are being exploited to increase yield.

These options are now under exploration to realize their potential in improving crop yields in many international teams though a number of major international projects, including the Bill and Melinda Gates Foundation funded C4 Rice (http://c4rice.irri.org/) and Realizing Improved Photosynthetic Efficiency (http://www.ripe.uiuc.edu) projects, the Center of Excellence for Photosynthesis Research (http://photosynthesis.org.au/). Though it is recognized that there are several targets and approaches to improve canopy photosynthesis, most of the current research focuses on photosynthetic efficiency at the leaf level, more efforts should be taken to explore the impacts of different engineering options on the canopy photosynthesis. In fact, there has been many reports regarding the lack of correlation between instantaneous measurements of leaf photosynthesis and crop yields (Evans and Dunstone 1970). Understanding limitations of canopy photosynthesis and identify leaf features that can confer higher canopy photosynthetic CO_2_ uptake rates are required to facilitate the application of photosynthesis in current crop breeding for higher yield potential (Zhu et al. 2010).

Canopy photosynthesis is inherently complex. It is influenced by a large number of factors, including physiological and architectural parameters, and external environmental conditions (Peng et al. 2000; Zhu et al. 2012). As a result, leaves inside a canopy each experience different biochemical limitations and these change over time. Dependent on the crop architecture, the growing location, planting density, planting direction, and leaf physiological parameters at different layers of a canopy, dramatic difference exists in the proportion of leaves limited by light absorption, electron transfer, RuBP regeneration, or Rubisco (Farquhar et al. 1980). As a result of this, the impacts of manipulating leaf photosynthetic properties on canopy photosynthesis are highly non‐linear and even Counter‐intuitive (Zhu et al. 2012). In recent years, there has been good progress in both the theoretical approach to modeling canopy photosynthesis and experimental methods to measure canopy photosynthesis, which promise to rapidly advance our ability to pinpoint the most effective approach to engineer canopy photosynthesis in a crop‐ and in a region‐specific manner. The purpose of this perspective paper is to discuss the current status on canopy photosynthesis from the perspective defining options to engineer improved canopy photosynthesis to support a region‐specific breeding. Specifically, we emphasize the potential factors that will influence the total canopy photosynthetic CO_2_ uptake rates, models and experimental approaches to quantify canopy photosynthesis and propose a new concept of model‐guided design of an ideal canopy for future crops. Readers can refer to (Zhu et al. 2012) about the heterogeneity of physiology inside a canopy and the overall structure and rationale for developing a new mechanistic dynamic systems model of canopy photosynthesis.

## Quantification of Canopy Photosynthesis

Quantification of canopy photosynthesis requires accurate estimation of photosynthetic rates of all leaves inside a canopy. This is inherently challenging because the microclimate inside a canopy, such as light, CO_2_, temperature, and humidity, is highly heterogeneous both spatially and temporally (Pearcy 1990; Zhu et al. 2004a, 2012; Song et al. 2013). As a result, leaves at different layers of a canopy normally experience different biochemical limitations. Leaves at the top of a canopy are usually light saturated; while leaves at the bottom layers of a canopy are usually light limited. The heterogeneity of light is also reflected in the appearance of sunflecks and shade‐flecks inside a canopy, which in turn is influenced by the canopy architectures and wind inside a canopy (Pearcy 1990). The physiological status of leaves at different layers of the canopy also vary dramatically (Evans and Poorter 2001; Niinemets 2007). On a broad scale, leaves at the top layers of a canopy are usually thicker, having higher chlorophyll *a:b* ratio, higher Rubisco concentration, etc. compared to the leaves at the lower layers (Terashima and Evans 1988; Hikosaka and Terashima 1995; Evans and Poorter 2001). Furthermore, plants adjust their metabolism to cope with different sunflecks and shade‐fleck patterns within the lower layers of a canopy. For example, understory plants usually have leaves that have much higher assimilatory charge, which enable plants to rapidly utilize the incoming sunflecks etc. (Pearcy 1990).

**Figure 1:**
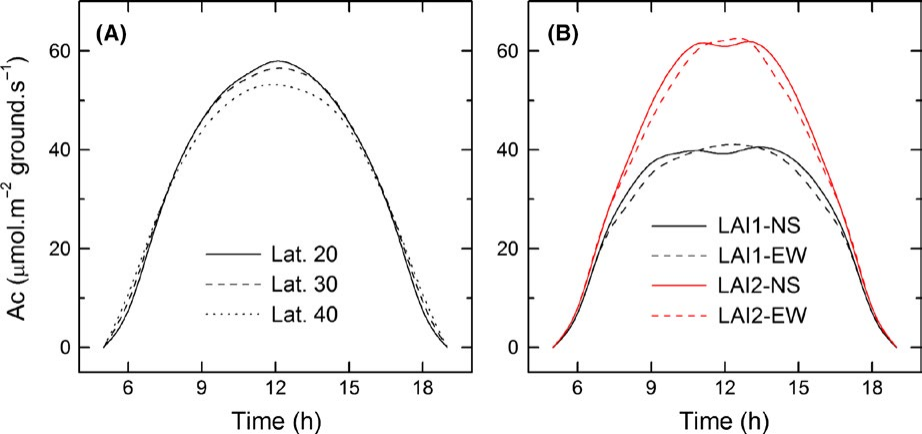
The impacts of varying growth location and growth direction on canopy photosynthetic rates. The simulation was conducted for a rice canopy based on methods for 3D canopy reconstruction (Song et al. 2013). (A) The impacts of varying the growth latitudes on the diurnal canopy photosynthetic rates; (B) The impacts of varying the leaf area index (LAI) and also the growth direction on the diurnal canopy photosynthesis. NS (North‐south direction); EW (East‐west direction).

Even though being inherently challenging, canopy photosynthesis has been modelled since the 1950s (Monsi and Saeki 1953). Different models, each with different degree of simplifications regarding the heterogeneity of microclimates inside the canopy, have been constructed in the past, see review by Zhu et al. (2012). Among these, the big leaf model made the assumption that the total canopy photosynthetic CO_2_ uptake rate can be effectively represented by a single leaf. Due to its simplicity, it has been used as a basic model in large‐scale general circulation model (GCM) (Sellers et al. 1996); however, the connection between this model with canopy architecture and physiological parameters are largely missing. The sunlit‐shaded model assumes that leaves in the canopy can be effectively classified as being either shaded leaf or sunlit leaf, each with an associated leaf area index. The leaf physiological parameters, such as maximal rate of RuBP and CO_2_ saturated rate of RuBP carboxylation (*V*_cmax_) and maximal rate of photosynthetic electron transfer (*J*_max_), can be effectively represented in such a model. Furthermore, the sunlit‐shaded model is relatively simple and easy to use (dePury and Farquhar 1997). As a result, it is used widely in the research community of photosynthesis physiology, ecology and agronomy. Recently, Song et al. (2013) developed a more mechanistic canopy photosynthesis model. This model can predict the detailed light environments inside a canopy by using realistic 3D reconstruction of a canopy with defined architecture combined with a forward ray tracing algorithm. The different physiological parameters for individual leaves can be incorporated into this model. As a result, the Song et al. (2013) enables evaluation of different architectural and physiological properties on canopy photosynthetic CO_2_ uptake rates. With this model, even the impacts of varying growth regions and planting density, planting direction on the total canopy photosynthetic CO_2_ uptake rates can be evaluated as well. Figure 1 shows that the growth location and planting direction greatly influence canopy photosynthetic rates. At a particular latitude of a particular growth region, the ideal canopy architecture for optimal canopy photosynthetic CO_2_ uptake henceforth should be defined. Different methods to directly measure canopy photosynthesis have been developed. The Bowen ratio/energy balance method is appropriate to quantify the total canopy gas exchange for a large area (Cellier and Olioso 1993). Canopy chamber approach, including both the open system chamber and the closed system chamber, has been developed to evaluate canopy photosynthesis at a plot scale (Dugas 1993; Dugas et al. 1997; Johnson et al. 2003; Song et al. 2016). These measurement systems hold great potential in model development and evaluation of germplasm to select lines with enhanced photosynthetic efficiencies.

## Canopy Photosynthesis and Crop Engineering and Breeding

In wheat, screening for improved leaf photosynthetic efficiency did not directly lead to enhanced canopy photosynthesis and crop yield and in fact the light saturated rate of leaf photosynthesis in wheat is negatively correlated with the leaf area index (Evans and Dunstone 1970). Increased leaf photosynthetic efficiency was gained by increasing leaf thickness, which unfortunately is correlated to decreased leaf area index (Evans and Dunstone 1970). However, the total canopy photosynthesis, rather than just leaf photosynthesis, is positively correlated with the biomass accumulation (Zelitch 1982). There are complex nonlinear interactions among crop architectural parameters and leaf physiological parameters, which jointly determine the optimal parameters to gain increased canopy photosynthetic CO_2_ uptake rate. This is clearly demonstrated by the impacts of different leaf area index on the potential gain of manipulation Rubisco kinetic properties on canopy CO_2_ uptake rates: at a higher leaf area index, there is increased benefit of engineering a Rubisco with higher specificity into a canopy (Zhu et al. 2004b). Similarly, it is expected that decreasing leaf chlorophyll concentration will have different consequences for canopies with different architecture (Ort et al. 2011). The increased leaf photosynthetic properties is usually associated with increased leaf chlorophyll concentrations, which can lead to altered light environments inside a canopy and hence altered canopy photosynthetic CO_2_ uptake rate as well. All these complex interactions necessitate application of detailed canopy systems models to design optimal parameters for enhanced canopy photosynthesis.

Over the last decade, a number of options to manipulate photosynthesis for enhanced photosynthetic efficiency have been identified. These different options were designed to overcome the limitation of photosynthesis at different biophysical or biochemical steps (Zhu et al. 2010; Long et al. 2015). For example, manipulation of leaf chlorophyll content mainly dealing with the excess energy at top layers and increase light availability at lower layers of a canopy; the potential impacts of this manipulation rely on not only leaf area index, canopy architecture, but also growth locations (Song 2004). Manipulation of the recovery from the photoprotective state aims to overcome the loss of quantum yield after plants are shifted from high light to low light (Zhu et al. 2004b). Engineering C_4_ photosynthesis into a C_3_ leaf aims to overcome the limitation of the Rubisco specificity factor on leaf photosynthetic rate under the current atmospheric CO_2_ conditions (Hibberd et al. 2008).

Considering that leaves at different locations of a canopy experience different microclimates and hence different biochemical limitations, it is important to design different crop ideal types with potentially different leaf photosynthetic properties for leaves at different layers of a canopy. A “smart canopy” concept has been proposed where the upper leaves should be more vertical, equipped with Rubisco with higher specificity, and smaller antenna size, as compared to lower layers of a canopy; furthermore, leaves at the lower layers of the canopy can be engineered to have enhanced chlorophyll d concentration to better fit the local light environments (Ort et al. 2015).

## Canopy Photosynthesis Underlines the Ideal‐type Breeding

**Figure 2:**
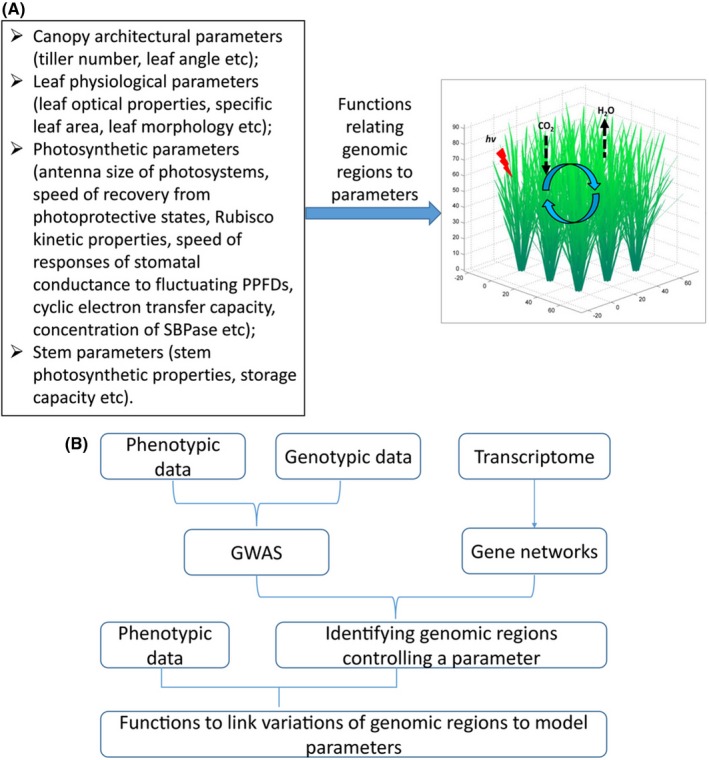
The parameters required for developing an idea type of a particular crop. (A) The general structure of the model. The model will incorporate both the detailed description of the canopy architectural parameters and also the detailed description of the photosynthetic processes. Functions relating genomic variations to variations of parameters will be used in the model so that the model can predict the consequences of different genetic variations on photosynthetic properties. (B) The procedure to establish the function to linking genetic variations to parameters used in the genetics‐based dynamic systems model of canopy photosynthesis.

What are the major components for the ideal‐type design from a canopy photosynthesis perspective? They include (1) the canopy architectural parameters, for example leaf length, leaf angle, leaf curvature, shape, leaf number, tiller number, planting density etc.; (2) physiological and biochemical parameters, which include leaf chlorophyll content, nitrogen content, content and activation state of key enzymes related to photosynthesis and parameters related to the recovery from the photoprotective state, parameters related to engagement of cyclic electron transfer, parameters related to stomatal responses, etc. The ideo‐type design is to identify optimal combination of these different parameters for a crop grown under a defined geological location (Fig. 2A). Though not guided by a mechanistic canopy photosynthesis model, ideotype‐guided breeding has been practiced for a long time. In fact, the concept of ideotype breeding was first proposed by Donald (1968). Since then, it has been widely practiced by breeders from both private and public sectors. Rice breeders in China and also in the International Rice Research Institute(IRRI) proposed various ideotypes for Japonica and indica, see review by Peng et al. (2008). For example, IRRI proposed the features of ideotype (or a new plant type) include low tillering number, few unproductive tillers, 200–250 grains per panicle, leaves that are thick, dark green, and erect, a plant height of 90–100 cm, thick and sturdy stems, vigorous root system, a growth duration of 100–130 days, and an increased harvest index (Peng et al. 1994). At IRRI, breeding based largely on these features with slight modifications has led to generation of many breeding lines and also the release of cultivars with increased yield potential (Peng et al. 2008). It is worth mentioning that most of the ideotype characteristics were determined based on computer simulations based on models with a simplified canopy architecture description. In China, Prof Longping Yuan proposed the features of rice ideotype, which include: moderate tillering capacity, heavy and drooping panicle at maturity, a plant height of 100 cm, panicle length of 60 cm at maturity, and specific features for the top three leaves, and a harvest index being above 0.55 (Yuan 2001). These ideotypes describe the morphology of the top three leaves, including their length, width, thickness, erectness, leaf angle, and also define the leaf area index (Yuan 2001). Most of these ideotype‐related features are related to canopy photosynthesis. Theoretically, different ideotypes should be developed for different growth regions or environments, as reflected in the difference in the ideotypes defined by IRRI and Prof. Yuan. From this perspective, the mechanistic model of canopy photosynthesis, which incorporates the detailed three‐dimensional canopy architectural parameters and physiological parameters of leaves at different locations, is needed to provide an objective and systematic method to tailor the features of ideotype to gain the optimal canopy photosynthesis and productivity for crops that are grown at defined locations.

## Toward a Genetics‐based Crop Systems Models to Guide Ideal‐type Design

As discussed earlier, the Song et al. (2013) model can be used to explore the optional canopy architecture, metabolic features, planting density, etc. for a crop at a particular geological location (Fig. 2A). However, to enable such a model to be used effectively in crop breeding, the direct linkage of the model parameters to its genetic basis needs to be established. In other words, the alleles or genes or molecular markers controlling each feature in the mechanistic crop systems model need to be defined. It is important to point out here that for a particular trait, such as leaf angle, there are often multiple different alleles controlling it (Wang and Li 2008). Depending on the various alleles in a particular rice accession, rice shows different leaf angles. Similarly, other parameters required for defining ideo‐type are also controlled by a number of alleles. Methods to link such genetic variations with parameters in systems model have been developed and used to predict a number of critical physiological and developmental parameters, such as flowering time (Reymond et al. 2003; van Eeuwijk et al. 2005; Yin et al. 2005). If a model with direct linkage between genetic variations and model parameters can be built, such a model can be immediately used to optimize allele combinations to gain maximal canopy photosynthetic rates at a particular location for a defined crop.

How far are we from realizing such a genetics‐based canopy photosynthesis model for one plant species, such as rice? Rice possibly represents the best studied crop species so far. Many functional relationships between allele variations and canopy architectural parameters have been established already for rice (Zuo and Li 2013). For other crops, such functional studies are much less established comparatively. For photosynthesis‐related parameters, so far, little is known about their association with allelic variations. Coordinated efforts are needed to establish new relations which can be used to predict photosynthetic parameters based on allelic contents and environmental conditions. The rapid advances in the modern phenomics facility and NGS technology are now offering an unprecedented opportunity to realize this. To do this, for each particular cultivar, using a large‐scale phenomics facility to measure the photosynthetic parameters under a diverse set of environmental conditions for a panel of genetically diverse accessions will be the first step (Lawson et al. 2012). This information, coupled with the genome wide association studies, QTL analysis, traditional genetics, and network inference approaches, can be used to identify the major alleles controlling photosynthetic efficiency under different environmental conditions (Fig. 2B). Once a genetics‐based systems model for rice is established, the same systems approach can be extended for all major food and energy crops to guide breeding and engineering for enhancing yields (Chu 2015; Long et al. 2015, Zhu et al. 2015).

**Figure 3:**
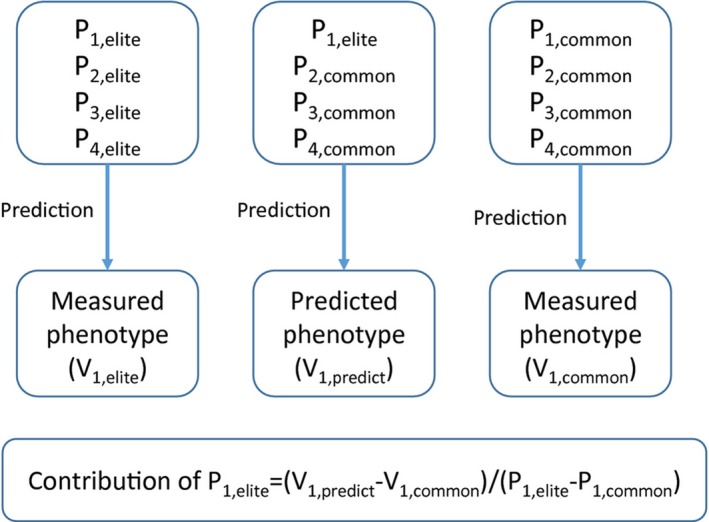
The routine for identifying the key parameters controlling the canopy photosynthetic light or nitrogen use efficiencies of a crop. P_1,elite_, P_2,elite,_ … … P_1,common,_ P_2,common_ are parameters for the elite or common cultivars. The V_1,elite_ or V_1,common_ are the predicted value of a particular phenotype. Synthetic cultivar is a hypothetical cultivar in which the value of parameter 1 from common cultivar (P_1,common_) is used to replace the parameter value of the elite cultivar (P_1,elite_).

There are two potential applications of using a genomics‐based model to guide the ideo‐type design. On one side, the genetics‐based model can be used to identify the most limiting step or parameter for light or nitrogen use efficiency for a particular crop grown at a particular region (Fig. 3). It can be used to identify optimal allele combinations to gain maximal CO_2_ uptake for a particular crop species. This optimal allelic combination can then be used to guide parental line selection and marker‐assisted breeding of new cultivars. On the other side, the genetics‐based model can be used to explore the best breeding or engineering strategy for a particular rice accession. In other words, by parameterizing such a genetics‐based model for a particular accession, we can use the model to identify the step exerting the highest control over canopy photosynthesis and further define the optimal allele to use to improve canopy photosynthetic efficiency in this particular rice line.

## Summary

The heterogeneity of microclimate inside a canopy requires using a mechanistic model of canopy photosynthesis to identify the optimal architectural and physiological parameters to support modern crop breeding or breeding. A mechanistic model of canopy photosynthesis is now available which enables one to evaluate impacts of manipulating canopy architectural and physiological parameters on canopy photosynthesis. The model can be used to define region‐specific optimal crop parameters and management practices. The challenge now is to develop a genetics‐based model of canopy photosynthesis by incorporating functional relationship between allelic variations and canopy parameters. Such a genetics‐based model holds great potential in guiding marker‐assisted breeding or genomic selection in the post‐genomics era.

## Conflict of Interest

None declared.
